# Serum 5-Hydroxyindoleacetic Acid Measurements for the Diagnosis and Follow-up of Carcinoid Syndrome

**DOI:** 10.1210/clinem/dgaf263

**Published:** 2025-05-02

**Authors:** Makarious Kerolles, Merijn C F Mulders, Mina Mirzaian, Sjoerd A A van den Berg, Richard A Feelders, Wouter W de Herder, Johannes Hofland

**Affiliations:** Department of Internal Medicine, Section of Endocrinology, ENETS Center of Excellence, Erasmus Medical Center Cancer Institute, 3015 GD Rotterdam, The Netherlands; Department of Internal Medicine, Section of Endocrinology, ENETS Center of Excellence, Erasmus Medical Center Cancer Institute, 3015 GD Rotterdam, The Netherlands; Department of Clinical Chemistry, Erasmus Medical Center, 3015 GD Rotterdam, The Netherlands; Department of Internal Medicine, Section of Endocrinology, ENETS Center of Excellence, Erasmus Medical Center Cancer Institute, 3015 GD Rotterdam, The Netherlands; Department of Clinical Chemistry, Erasmus Medical Center, 3015 GD Rotterdam, The Netherlands; Department of Internal Medicine, Section of Endocrinology, ENETS Center of Excellence, Erasmus Medical Center Cancer Institute, 3015 GD Rotterdam, The Netherlands; Department of Endocrinology, Diabetes and Metabolism, New York University Langone Medical Center, New York, NY 10017, USA; Department of Internal Medicine, Section of Endocrinology, ENETS Center of Excellence, Erasmus Medical Center Cancer Institute, 3015 GD Rotterdam, The Netherlands; Department of Internal Medicine, Section of Endocrinology, ENETS Center of Excellence, Erasmus Medical Center Cancer Institute, 3015 GD Rotterdam, The Netherlands

**Keywords:** 5-hydroxyindoleacetic acid, carcinoid syndrome, neuroendocrine tumor, SSA, biomarker

## Abstract

**Context:**

The biochemical diagnosis of carcinoid syndrome (CS) is established through the measurement of 24-hour urine 5-hydroxyindoleacetic acid (5-HIAA), but these measurements are prone to sampling error and may be troublesome for patients. Serum 5-HIAA measurements might constitute a more reliable and convenient alternative to diagnose CS.

**Objective:**

To assess the diagnostic value of serum 5-HIAA measurements in patients with CS.

**Design:**

Retrospective cohort study.

**Setting:**

Tertiary care hospital.

**Patients:**

379 patients with a neuroendocrine tumor (NET), of whom 136 (35.9%) had CS; 153 control samples were included.

**Intervention:**

Paired serum and 24-hour urine 5-HIAA measurements.

**Main Outcome Measure(s):**

Performance of serum and 24-hour urine 5-HIAA for the diagnosis of CS, measured by area under the receiver operating characteristic curve (AUROC).

**Results:**

Serum 5-HIAA performance was similar to that of 24-hour urine 5-HIAA for the diagnosis of CS in the total NET cohort (n = 379, AUROC 0.824 vs 0.843, *P* = .50) and in a subgroup of somatostatin analog (SSA)-naïve patients (n = 141, AUROC 0.915 vs 0.938, *P* = .66). Optimal cutoff value of serum 5-HIAA for the diagnosis of CS was 139.4 nmol/L (sensitivity 96.3%, specificity 87.6%) as determined in a subgroup analysis of SSA-naive patients with CS and controls. Serum 5-HIAA correlated well with 24-hour urine 5-HIAA (r = 0.892, *P* < .001) and the presence of flushing, diarrhea, and carcinoid heart disease (odds ratio 1.047-1.073 for every 100 nmol/L increase, *P* < .001).

**Conclusion:**

Serum 5-HIAA measurements are equivalent to 24-hour urine 5-HIAA measurements for the diagnosis of CS in patients with NET and form an accessible alternative.

Neuroendocrine neoplasms (NENs) form a heterogenous group of tumors arising from neuroendocrine cells dispersed throughout the body. NENs can be categorized into well-differentiated neuroendocrine tumors (NETs) and poorly differentiated NETs and neuroendocrine carcinomas and are becoming increasingly relevant due to their rising incidence and prevalence (1, 2). In the United States and the United Kingdom, the incidence of NENs increased 6.4-fold (1973-2012: 1.09 to 6.98 per 100.000 inhabitants) and 3.7-fold (1995-2018: 2.35 to 8.61 per 100.000 inhabitants), respectively, over the past decades (1, 2).

Carcinoid syndrome (CS) is the most frequently encountered hormonal syndrome in patients with a metastatic NET, in particular of midgut origin, occurring in approximately 20% of cases (3). CS is characterized by symptoms of flushing and diarrhea in the presence of elevated circulating levels of serotonin or its main metabolite 5-hydroxyindoleacetic acid (5-HIAA) (4). Other less prevalent symptoms include wheezing and fibrotic complications like carcinoid heart disease (CHD) and mesenteric fibrosis (5, 6). Patients with CS have a shorter overall survival and a reduced quality of life compared to NEN patients without CS (3, 7, 8).

The main metabolite of serotonin, 5-HIAA, is used to biochemically assess the presence of CS. At present, measuring 5-HIAA in 24-hour urine is recommended for the diagnosis and follow-up of CS (4). However, despite an acceptable sensitivity of 77% and a specificity of 97% (9), 24-hour urine 5-HIAA measurements are prone to sampling errors due to incomplete collection of urine over a 24-hour period. Also, 24-hour urine 5-HIAA measurements are inconvenient for patients. First, a 48- to 72-hour tryptophan- and serotonin-free diet is generally advised before collecting 24-hour urine 5-HIAA (10), as dietary tryptophan and serotonin intake might increase the 24-hour urine 5-HIAA values, possibly leading to false-positive results (11-14). Second, the intraindividual variation of 24-hour urine 5-HIAA measurements necessitates the collection of 24-hour urine for 2 consecutive days (4, 15). Third, patients with CS often suffer from frequent diarrhea, complicating urine collection.

Serum 5-HIAA (16-18) and plasma 5-HIAA (9, 19-21) measurements both seem to correlate well with 24-hour urine 5-HIAA measurements. Previous studies assessing the value of either serum or plasma 5-HIAA for the diagnosis of CS found sensitivities (61-91%) and specificities (62-100%) similar to 24-hour urine 5-HIAA (9, 17, 19, 22). However, these studies only included 57 to 97 patients (9, 17, 19) with only 1 study reporting data on serum 5-HIAA for the diagnosis of CS (22), raising the need for a large confirmative study. Additionally, only 1 study reports data on the value of plasma 5-HIAA during the follow-up of patients with CS. In this study, we aimed to assess the value of serum 5-HIAA measurements as a more reliable and convenient alternative to 24-hour urine 5-HIAA in the diagnosis and follow-up of CS.

## Methods

### Study Population

379 patients with a NET, diagnosed through histology or somatostatin receptor imaging, and at least 1 paired serum 5-HIAA and 24-hour urine 5-HIAA measurement between 2021 and 2023 at the Erasmus MC ENETS Center of Excellence, were included. CS was present in 136 out of 379 patients (35.9%). Of the total of 141 patients who were somatostatin analogs (SSA)-naïve, 38 had CS. 243 NET patients without CS formed a control NET group; of these patients, a subgroup of 103 cases were SSA-naïve. Additionally, a separate non-NET control group consisted of 153 patients without suspicion of a NET. Patients were not instructed to adhere to a tryptophan- or serotonin-restricted diet beforehand. Serum and 24-hour urine 5-HIAA measurements were considered paired when the time interval was less than 6 months and no relevant locoregional or systemic therapy [SSA, peptide receptor radionuclide therapy with ^177^Lu-DOTATATE (PRRT)] was initiated in between. The pairs with the shortest time interval were chosen if multiple pairs were possible. A mean 24-hour urine 5-HIAA was calculated if 2 24-hour urine 5-HIAA measurements were available. Data were collected from electronic patient files. Serum 5-HIAA was measured within 4 hours after sample collection. For this retrospective cohort study, the need for written informed consent was waived by the Medical Ethics Committee of the Erasmus MC (MEC-2024-0044).

### Outcome Measures

The primary endpoint of this study was the diagnostic performance of serum 5-HIAA and 24-hour urine 5-HIAA as markers for CS. CS was defined as the presence of diarrhea and/or flushing and/or CHD combined with an elevated urinary 5-HIAA level (>50 µmol/24 hours), in accordance with the 2022 ENETS guidance paper for CS (4). Secondary outcomes were patient and tumor characteristics, the optimal cutoff value of serum and 24-hour urine 5-HIAA for the diagnosis of CS, and the change in serum 5-HIAA after treatment with SSA. Serum 5-HIAA and 24-hour urine 5-HIAA values within 6 months before the start of SSA and within 6 months after the start of SSA treatment were considered paired. Also, the association between serum and 24-hour urine 5-HIAA, the presence of CS-related symptoms, and patient and tumor characteristics was investigated, which were recorded at the time of the first paired measurement.

### Laboratory Analysis

All laboratory analyses were performed in the clinical chemistry laboratory of the Erasmus MC. The analysis of 5-HIAA in serum samples was conducted using reversed-phase ultraperformance liquid chromatography tandem mass spectrometry (UPLC-MS/MS) (Waters Xevo-TQ-S Micro, Waters, Etten-Leur, The Netherlands). Serum 5-HIAA values above the upper limit of the measurement range were recorded as 8000 nmol/L. The analysis of 5-HIAA in 24-hour urine samples collected up to August 2022 was conducted using reversed-phase high-performance liquid chromatography with fluorometric detection. From August 2022, this analysis was conducted using reversed-phase UPLC-MS/MS (Waters Xevo-TQ-S Micro, Waters, Etten-Leur, The Netherlands). The upper limit of normal of 50 μmol/24 hours was used as cutoff value for 24-hour urine 5-HIAA (4, 23).

### Statistical Analysis

Normally distributed data are expressed as median with SD, nonnormally distributed data are expressed as median with interquartile range (IQR) and categorical data are expressed as frequencies (%). Patients with missing values were excluded in the respective analysis. A receiver operator characteristics analysis was performed to assess the diagnostic performance of serum 5-HIAA and 24-hour urine 5-HIAA for the diagnosis of CS. Comparisons were made between SSA-naïve patients with CS and both non-NET controls and patients with NET without CS and between all included patients with CS and both non-NET controls and all included patients with NET without CS. For sensitivities and specificities, a 95% confidence interval (CI) was calculated using the modified Wald method (24). The resulting areas under the ROC curve (AUROC) were compared using the Hanley and McNeil method (25). Optimal cutoff values for serum 5-HIAA and 24-hour urine 5-HIAA were determined using the Youden index (26). Pearson correlation was performed of serum 5-HIAA and 24-hour urine 5-HIAA values after normal distribution was reached by log_10_ transformation. Paired nonnormally distributed data was analyzed using a Wilcoxon signed-rank test. Nonpaired nonnormally distributed data was analyzed using a Mann-Whitney U test. Logistic regression analyses were performed to assess the association between the individual CS-related symptoms and serum or 24-hour urine 5-HIAA and patient, tumor, and laboratory characteristics. Linear regression analysis was performed to assess the association between serum or 24-hour urine 5-HIAA and patient and tumor characteristics. The potential influence of estimated glomerular filtration rate (eGFR) on serum and 24-hour urine 5-HIAA levels was assessed using a Spearman correlation.

Statistical analyses were performed using SPSS (version 28, IBM Corp.). Comparison of AUROC values was performed using MedCalc (version 22.014, Medcalc Software Ltd.). A 2-tailed *P*-value of .05 was considered statistically significant. Bonferroni correction for multiple testing was applied to the linear regression results.

## Results

### Patient Characteristics

A total of 379 patients with NET with at least 1 paired serum and 24-hour urine 5-HIAA measurement were included (Table 1). Median age at the time of the first paired measurement was 68 years (IQR 61-74 years), and 210 patients (55%) were male. The primary tumor originated from the midgut in the majority of the patients with a NET (74.4%). In patients with CS, the NET originated from the midgut in 81.6% and from the lung in 3.7%, while 11% had an unknown primary tumor.

**Table 1.** Baseline characteristics of the included patients

Patient characteristics	Total NET cohort (n = 379)
Sex, male, n (%)	210 (55.4)
Age, years, median (IQR)	68 (61-74)
Primary tumor location, n (%)	
Lung	18 (4.7)
Gastroduodenal	3 (0.8)
Pancreas	29 (7.7)
Midgut	282 (74.4)
Hindgut	5 (1.3)
Other	7 (1.8)
Unknown	35 (9.2)
Tumor grade, n (%)	
Grade 1	180 (47.5)
Grade 2	156 (41.2)
Grade 3	8 (2.1)
Unknown	35 (9.2)
Regional lymph node metastasis, n (%)	
Yes	302 (79.7)
No	65 (17.2)
Unknown	12 (3.2)
Distant metastasis, n (%)	
Yes	324 (85.5)
Liver	280 (73.9)
Nonregional lymph nodes	180 (47.5)
Bone	150 (39.6)
Lung	24 (6.3)
Other	68 (17.9)
No	47 (12.4)
Unknown	8 (2.1)
Carcinoid syndrome, n (%)	
Yes	136 (35.9)
Carcinoid heart disease	27 (7.2)*^a^*
No	243 (64.1)
Laboratory characteristics, median (IQR)	
Serum 5-HIAA, nmol/L	367.6 (102.5-1333.1)
Urine 5-HIAA, μmol/24h	111.7 (45.6-374.3)
Chromogranin A, μg/L	367.5 (119.5-1131.5)*^b^*
Alkaline phosphatase, U/L	96 (77-140.3)*^c^*
eGFR, mL/min/1,73 m^2^	66.9 (52.1-87)*^d^*
Prior treatment, n (%)	
Primary tumor surgery	137 (36.1)
Liver-directed therapy	14 (3.7)
SSA	238 (62.8)
PRRT	43 (11.3)
Everolimus	6 (1.6)

Abbreviations: 5-HIAA, 5-hydroxyindoleacetic acid; eGFR, estimated glomerular filtration rate; IQR, interquartile range; NET, neuroendocrine tumor; PRRT, peptide receptor radionuclide therapy with ^177^Lu-DOTATATE; SSA, somatostatin analog.

^*a*^Missing cases: n = 6.

^*b*^Missing cases: n = 29.

^*c*^Missing cases: n = 37.

^*d*^Missing cases: n = 8.

Tumors were grade 1 in 180 cases (47.5%) and grade 2 in 156 cases (41.2%). Liver metastases were present in 280 patients (73.9%). Respectively, 238 (62.8%) and 43 (11.3%) of patients had received treatment with SSA or PRRT prior to the first paired serum measurement. The median time interval between serum and 24-hour urine 5-HIAA measurements was 0.1 days (IQR 0-11 days). In all patients with a NET, median serum 5-HIAA was 367.6 nmol/L (IQR 102.5-1333.1 nmol/L) and median 24-hour urine 5-HIAA was 111.7 μmol/24 hours (IQR 45.6-374.3 μmol/24 hours). In patients with CS, median serum 5-HIAA was 1327.1 nmol/L (IQR 503.4-3556.0 nmol/L), while patients without CS showed a median serum 5-HIAA value of 166.2 nmol/L (IQR 81.1-554.8 nmol/L). Within the entire cohort, data on CHD was present in 373 patients with a NET. CHD was present in 27 patients (7.2%). Patients with CHD showed a higher median serum 5-HIAA value compared to patients without CHD (3044.5 vs 308.2 nmol/L, *P* < .001). Patients with metastatic disease had a higher median serum 5-HIAA level compared to patients with localized disease (498.4 vs 93.8 nmol/L, *P* < .001).

**Figure 1.**
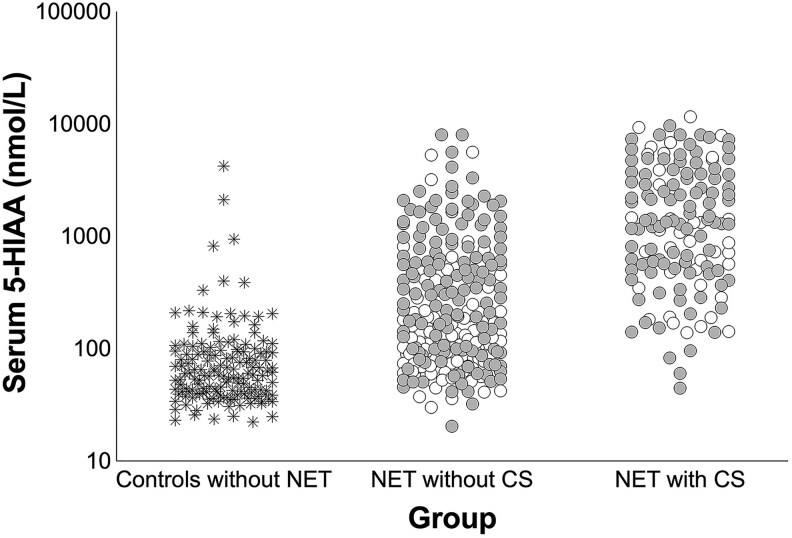
Distribution of serum 5-HIAA values in the included groups. Distribution of serum 5-HIAA values of the included controls without NET suspicion (n = 153), NET patients without CS (n = 243), and NET patients with CS (n = 136). The asterisks represent the controls without NET suspicion. The white dots represent the SSA-naïve patients, while the gray dots represent patients with prior SSA usage. Abbreviations: 5-HIAA, 5-hydroxyindoleacetic acid; CS, carcinoid syndrome; NET, neuroendocrine tumor; SSA, somatostatin analog.

153 serum 5-HIAA samples of controls were included. The median age of this cohort was 52 years (IQR 31-69), and 75 patients (49%) were male. Median serum 5-HIAA was 57.9 nmol/L (IQR 40.0-94.3 nmol/L). Distribution of serum 5-HIAA values in the control group without NET suspicion, NET patients without CS, and patients with CS are shown in Fig. 1.

### Diagnostic Performance

**Figure 2.**
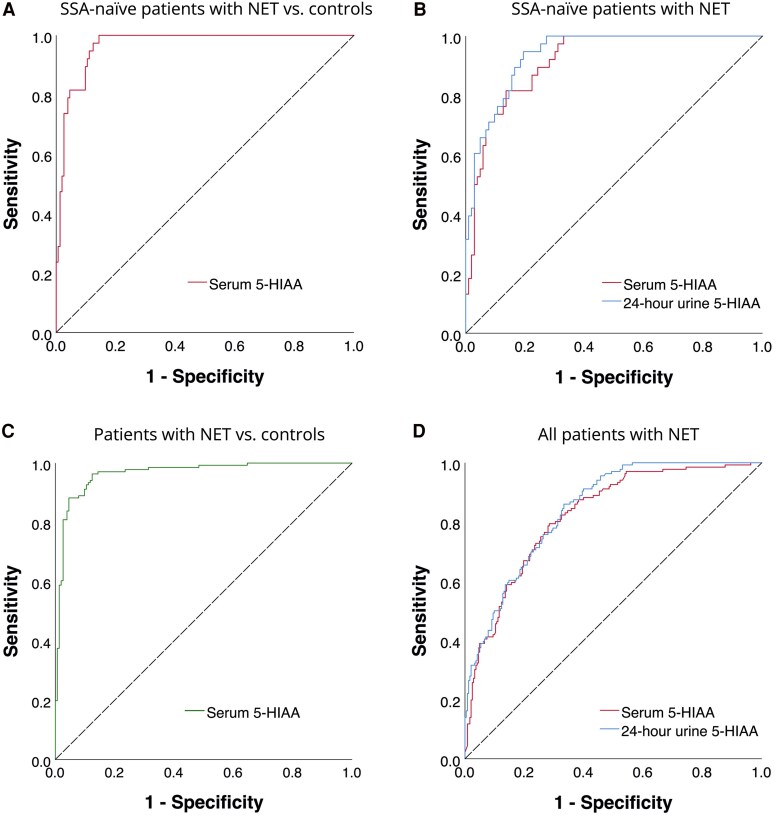
ROC curves for the diagnosis of carcinoid syndrome: (A) serum 5-HIAA in SSA-naïve patients with a NET using a control group without a NET (n = 191), (B) serum and 24-hour urine 5-HIAA in SSA-naïve patients with a NET (n = 141), (C) serum 5-HIAA in all patients with a NET using a control group without a NET (n = 289), (D) serum and 24-hour urine 5-HIAA in all patients with a NET (n = 379). Abbreviations: 5-HIAA, 5-hydroxyindoleacetic acid; NET, neuroendocrine tumor; ROC, receiver operating characteristics; SSA, somatostatin analog.

Serum 5-HIAA differentiates between SSA-naïve patients with CS (n = 38) and non-NET controls (n = 153) with an AUROC of 0.967 (95% CI: 0.945-0.989) (Fig. 2A). The optimal serum 5-HIAA cutoff value to diagnose CS was 139.4 nmol/L, which was accompanied by a sensitivity of 96.3% (95% CI: 91.5-98.7%) and a specificity of 87.6% (95% CI: 81.3-92.0%). Serum 5-HIAA values exceeded this cutoff value in 34/103 (33.0%) SSA-naïve NET patients without CS.

Serum and 24-hour urine 5-HIAA differentiate between SSA-naïve patients with CS (n = 38) and SSA-naïve patients without CS (n = 103) with AUROCs of 0.915 (95% CI: 0.870-0.961) and 0.938 (95% CI: 0.902-0.975), respectively, with no significant difference in diagnostic performance (*P* = 0.66) (Fig. 2B). In this analysis, using a control group of SSA-naïve patients with a NET, a higher optimal cutoff value of 283.4 nmol/L for the diagnosis of CS was found, with a sensitivity of 81.5% (95% CI: 66.3-91.1%) and a specificity of 86.4% (95% CI: 78.4-91.9%).

Comparing the total cohort of patients with CS (n = 136), which included patients on current SSA therapy, with serum samples of non-NET controls (n = 153), the AUROC of serum 5-HIAA to diagnose CS was 0.965 (95% CI: 0.945-0.985) (Fig. 2C). Comparing the total cohort of patients with CS (n = 136) with the total cohort of patients without CS (n = 243), the AUROCs for the diagnosis of CS were 0.824 (95% CI: 0.782-0.866) and 0.843 (95% CI: 0.806-0.881) for serum 5-HIAA and 24-hour urine 5-HIAA, respectively (Fig. 2D). The diagnostic performance did not differ between these 2 measures (*P* = .50).

Within the total cohort of patients with a NET with data available on the presence of CHD (n = 373), the AUROC for the diagnosis of CHD was 0.833 (95% CI: 0.836-0.930). In this analysis, a serum 5-HIAA optimal cutoff value of 1005.3 nmol/L was found for the diagnosis of CHD, which was accompanied by a sensitivity of 92.6% (95% CI: 75.5-99.0%) and a specificity of 74.8% (95% CI: 70.0-79.1%).

24-hour urine 5-HIAA showed a sensitivity of 100% (reference) and a specificity 68.9% (95% CI: 59.4-77.1%) for a cutoff value of 50 μmol/24 hours. In the subgroup analysis of SSA-naïve patients, the optimal cutoff for 24-hour urine 5-HIAA was 65 μmol/24 hours, with a sensitivity of 94.7% (95% CI: 81.8-99.5%) and a specificity of 80.6% (95% CI: 71.8-81.1%).

### Correlation Between Markers and Correlation With CS-related Symptoms

**Figure 3.**
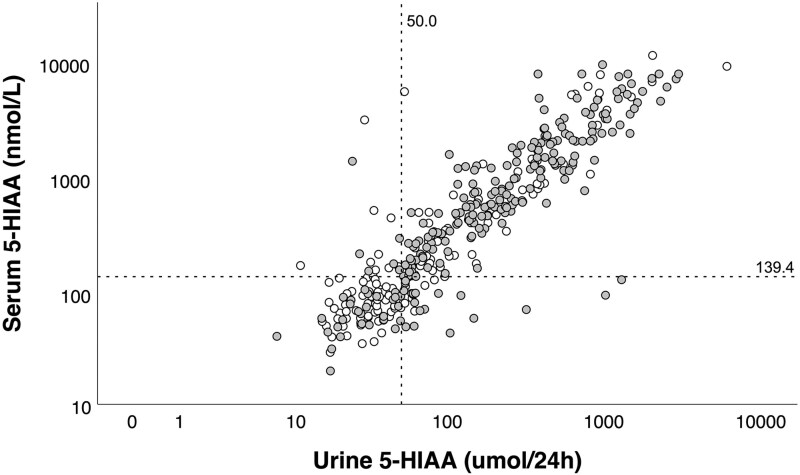
Correlation between serum and 24-hour urine 5-HIAA levels. Scatterplot of 379 patients with a NET with a paired serum and urine 24-hour urine 5-HIAA measurement. Log_10_ serum 5-HIAA is plotted against log_10_ 24-hour urine 5-HIAA. The white dots represent the SSA-naïve patients, while the gray dots represent patients with prior SSA usage. The y-axis reference line represents the serum 5-HIAA optimal cutoff of 139.4 nmol/L, while the x-axis reference line represents the 24-hour urine 5-HIAA cutoff of 50 μmol/24 hours. Abbreviations: 5-HIAA, 5-hydroxyindoleacetic acid; NET, neuroendocrine tumor; SSA, somatostatin analog.

Serum 5-HIAA levels showed a strong correlation with 24-hour urine 5-HIAA levels (r = 0.892, *P* < .001) (Fig. 3). This correlation was similar in SSA-naïve patients (r = 0.877, *P* < .001). Using the cutoff values of 139.4 nmol/L and 50 μmol/24 hours for serum and urine 5-HIAA measurements, respectively, 41/379 (10.8%) patients showed discrepant test results. Elevated serum 5-HIAA concentrations combined with normal 24-hour urine 5-HIAA test results were present in 16/379 (4.2%) patients, whereas the opposite results were also observed in 25/379 (6.6%) patients (Fig. 3). The majority of these patients (15 and 20, respectively) did not have CS-related symptoms.

Data regarding the presence of flushing, diarrhea, wheezing, and CHD were available in 341, 358, 264, and 373 patients, respectively. Flushing and diarrhea were the most frequently reported CS-related symptoms occurring in 104/341 (30.5%) and 73/358 (20.4%) of the total cohort and in 99/129 (76.7%) and 73/132 (55.3%) of the CS cohort, respectively. Wheezing and CHD were reported to a lesser extent, respectively occurring in 2/87 (2.3%) and 27/133 (20.3%) of patients with CS. Increases of serum and 24-hour urine 5-HIAA levels were both associated with the presence of CS-related symptoms (*P* < .001) (Table 2). Each 100 nmol/L increase in serum 5-HIAA level yielded a 5.1%, 4.7%, and 7.3% greater odds of the presence of flushing, diarrhea, and CHD, respectively. For urine 5-HIAA levels, each 100 μmol/24 hours increase yielded a 33.8%, 25.7%, and 31.5% greater odds of the presence of flushing, diarrhea, and CHD, respectively.

**Table 2.** Correlation of serum or urinary 5-HIAA concentrations with CS-related symptoms

CS-related symptoms	Serum 5-HIAA		24-hour urine 5-HIAA	
	OR (95% CI)*^a^*	*P*-value	OR (95% CI)*^b^*	*P*-value
Flushing***^c^***	1.0514 (1.0512-1.0516)	<.001^*f*^	1.3383 (1.3370-1.3397)	<.001^*f*^
Diarrhea***^d^***	1.0469 (1.0467-1.0471)	<.001^*f*^	1.2576 (1.2565-1.2587)	<.001^*f*^
CHD***^e^***	1.0728 (1.0725-1.0731)	<.001^*f*^	1.3151 (1.3137-1.3166)	<.001^*f*^

Abbreviations: 5-HIAA, 5-hydroxyindoleacetic acid; CHD, carcinoid heart disease; CI, confidence interval; CS, carcinoid syndrome; OR, odds ratio.

^*a*^ORs are shown for a 100 nmol/L increase in serum 5-HIAA.

^*b*^ORs are shown for a 100 μmol/24 hours increase in urine 5-HIAA.

^*c*^Missing cases: n = 38.

^*d*^Missing cases: n = 21.

^*e*^Missing cases: n = 6.

^*f*^ *P*-values are considered significant.

### Correlation With Patient and Tumor Characteristics

**Figure 4.**
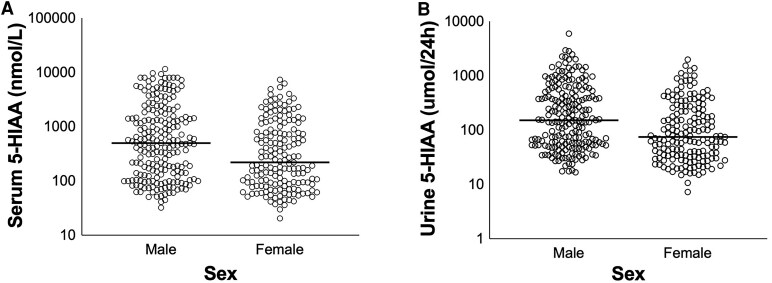
Serum and 24-hour urine 5-HIAA levels in male and female patients with a NET. Scatterplot of serum (A) and 24-hour urine 5-HIAA (B) values in male (n = 210) and female (n = 169) patients with a NET. The horizontal lines represent the median in the respective groups. Abbreviations: 5-HIAA, 5-hydroxyindoleacetic acid; NET, neuroendocrine tumor.

Multivariate linear regression revealed sex and chromogranin A as factors associated with serum and 24-hour urine 5-HIAA levels (Table 3). Male patients with a NET showed increased serum 5-HIAA (β 630.05, *P* = .007) and 24-hour urine 5-HIAA (β 173.85, *P* = .003) levels compared to female patients (Fig. 4). Chromogranin A levels showed a positive correlation with both serum 5-HIAA (n = 362, β 0.04, *P* < .001) and 24-hour urine 5-HIAA (n = 362, β 0.011, *P* < .001) values. No association between either Ki67 or alkaline phosphatase and serum or 24-hour urine 5-HIAA was observed.

**Table 3.** Correlation of serum and urinary 5-HIAA concentrations with patient and tumor characteristics

Characteristic	Serum 5-HIAA*^a^*		24-hour urine 5-HIAA*^b^*	
	β (95% CI)	*P*-value	β (95% CI)	*P*-value
Sex				
Female	Reference		Reference	
Male	630.05 (173.98-1086.11)	.007^*f*^	173.85 (60.93-286.77)	.003^*f*^
Ki67***^c^***	2.45 (−38.05-42.96)	.905	3.81 (−6.22-13.83)	.456
Chromogranin A (μg/L)***^d^***	0.04 (.03-.06)	<.001^*f*^	0.011 (.007-.015)	<.001^*f*^
Alkaline phosphatase (U/L)***^e^***	1.10 (−.55-2.74)	.191	0.34 (−.07-.75)	.099

Abbreviations: 5-HIAA, 5-hydroxyindoleacetic acid; CI, confidence interval.

^*a*^R^2^ = 0.157; *P* < 0.001.

^*b*^R^2^ = 0.194; *P* < 0.001.

^*c*^Missing cases: n = 74.

^*d*^Missing cases: n = 29.

^*e*^Missing cases: n = 37.

^*f*^ *P*-values are considered significant.

In addition, eGFR was negatively correlated with both serum 5-HIAA (n = 371, r = −0.281, *P* < .001) and 24-hour urine 5-HIAA values (n = 371, r = −0.152, *P* < .001). Due to the limited correlation coefficient, eGFR was not included in the linear regression analysis.

### Effect of SSA

**Figure 5.**
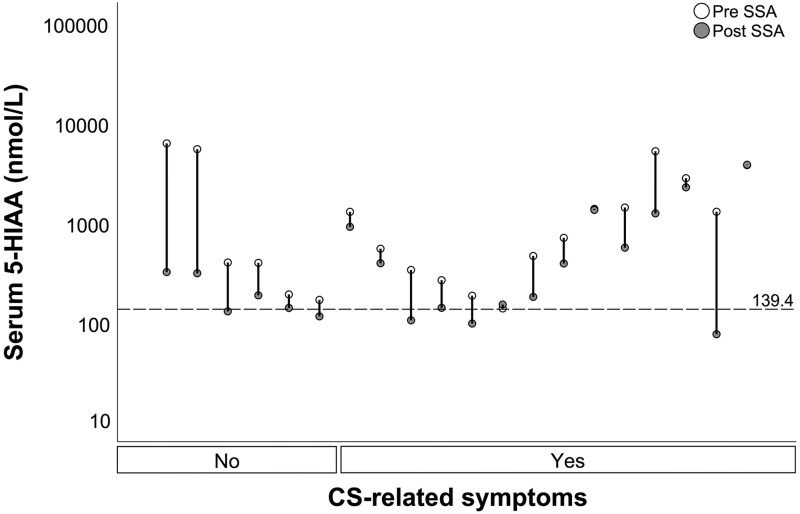
Effect of SSA on serum 5-HIAA. Before-after plot displaying individual serum 5-HIAA values before (white dots) and after (gray dots) the initiation of SSA treatment in patients with a NET and serum 5-HIAA values exceeding the found optimal cutoff value of 139.4 nmol/L (n = 21). The dashed reference line resembles the found optimal cutoff value of 139.4 nmol/L. Abbreviations: 5-HIAA, 5-hydroxyindoleacetic acid; CS, carcinoid syndrome; SSA, somatostatin analog.

In 20 patients with a NET and serum 5-HIAA values exceeding 139.4 nmol/L, values were available before and after the start of SSA treatment. The median time interval between the initiation of SSA treatment and the subsequent serum 5-HIAA measurement was 107 days (IQR 84-161 days). Serum 5-HIAA concentrations decreased from a median of 646.6 nmol/L to 256.8 nmol/L after the start of SSA treatment (*P* < .001) (Fig. 5).

## Discussion

Our findings indicate that serum 5-HIAA levels have a comparable performance to 24-hour urine 5-HIAA levels for the diagnosis of CS. A serum 5-HIAA optimal cutoff value of 139.4 nmol/L for the diagnosis of CS was established. Additionally, serum 5-HIAA levels showed a strong correlation with 24-hour urine 5-HIAA levels and with the presence of CS-related symptoms of flushing, diarrhea, and CHD.

Our findings are in line with previous prospective studies showing similarities between either serum or plasma and 24-hour urine 5-HIAA levels for the diagnosis of CS (9, 17, 19, 22). Only Ewang-Emukowhate and colleagues (22) report data on the diagnostic performance of serum and 24-hour urine 5-HIAA for the diagnosis of CS, finding a comparable diagnostic performance with AUROCs of 0.902 and 0.919, consistent with the findings of our study, respectively. Only Carling and colleagues (9) statistically tested the difference in diagnostic performance between plasma and 24-hour urine 5-HIAA, showing no significant difference (AUROC 0.902 vs 0.895, *P* = .89), which is consistent with our findings for serum 5-HIAA. Control groups in these studies consisted of either patients with irritable bowel syndrome or healthy controls presenting with symptoms suggestive of CS. The observed similarities in diagnostic performance of serum 5-HIAA and 24-hour urine 5-HIAA are further substantiated by the strong correlation between serum and 24-hour urinary 5-HIAA measurements among patients with NET found in this study, which is in accordance with previous literature (16, 17). Moreover, serum 5-HIAA showed an adequate diagnostic capacity for the diagnosis of CS, with an AUROC of 0.824 among patients with NET and an AUROC of 0.965 using a control group of patients without NET suspicion. This is comparable to the previously described studies investigating either serum or plasma 5-HIAA levels for the diagnosis of CS, showing a similar diagnostic capacity with AUROC ranging from 0.697 to 0.917 (9, 17, 19, 22). De Mestier and colleagues (19) found a lower AUROC of 0.697 for plasma 5-HIAA in their prospective cohort. This may be explained by the greater presence of SSA use in their cohort [68/80 (85%) vs 238/379 (62.8%) in our cohort], a factor that substantially influenced diagnostic performance in our study.

Using a control group of patients without NET suspicion, we found an optimal cutoff value of 139.4 nmol/L for diagnosing CS using serum 5-HIAA. This is comparable to the cutoff value of 135 nmol/L reported by Ewang-Emukowhate and colleagues (22), who also evaluated the diagnostic performance of serum 5-HIAA with a control group of patients without NET suspicion. Using a comparator group of SSA-naïve patients, we detected a higher optimal cutoff of 283.4 nmol/L to assess the presence of CS among patients with a NET compared to the optimal cutoff needed to distinguish patients with CS from NET patients without CS. This higher cutoff value might be explained by higher baseline serum 5-HIAA levels in patients with a NET compared to controls as previous studies have shown that serum 5-HIAA levels exceeding the upper limit of normal of 123 nmol/L effectively discriminate patients with NET from controls, irrespective of the presence of CS (16, 18, 27). These results suggest the use of cutoff values depending on the clinical situation as described earlier by Meijer and colleagues (28). Among patients with a NET presenting with CS-related symptoms, a higher serum 5-HIAA cutoff value might be appropriate for diagnosing CS compared to the cutoff value needed for diagnosing CS among patients without a NET history presenting with CS-related symptoms. However, the retrospective nature of this study raises uncertainty about the true absence of CS-related symptoms in SSA-naïve controls with a NET and elevated 24-hour urine 5-HIAA values, possibly leading to an overestimation of the serum 5-HIAA cutoff value of 283.4 nmol/L. We therefore suggest the use of the cutoff value of 139.4 nmol/L for diagnosing CS using serum 5-HIAA. We additionally found a serum 5-HIAA optimal cutoff value of 1005.3 nmol/L for the diagnosis of CHD. This is higher than the previously published cutoff value of 757 nmol/L as described by Lecoeur and colleagues (29) and may be explained by the greater presence of SSA use in their cohort.

Considering the usability of serum 5-HIAA in the follow-up of patients with CS, our study shows a strong correlation between serum 5-HIAA and the presence of CS-related symptoms of flushing, diarrhea, and CHD, as well as tumor burden as measured by chromogranin A. The correlation of serum 5-HIAA with tumor burden aligns with the findings of previous studies showing a correlation between chromogranin A and serum 5-HIAA in 112 patients with a NET (18). This was further confirmed by Wedin and colleagues (16), who described an association between serum 5-HIAA levels and liver tumor burden on computed tomography/magnetic resonance imaging assessments in 48 patients with NET. Furthermore, a negative correlation was found between eGFR and serum 5-HIAA compared to the results of Adaway and colleagues (17). This difference might be due to the limited number of patients with an impaired eGFR in this cohort. Nevertheless, given the findings of Adaway and colleagues (17), renal function should be considered when interpreting serum 5-HIAA values. Interestingly, female patients with a NET were found to have lower levels of both serum and 24-hour urine 5-HIAA when compared to male patients with NET. Only Tohmola and colleagues (18) assessed sex-related differences in 5-HIAA values in patients with a NET, finding no significant difference in serum 5-HIAA values. However, multiple previous studies have reported this sex-related difference for plasma 5-HIAA in patients with irritable bowel syndrome and in healthy controls (30-32). These findings suggest a reduced tryptophan input in women, which could be consistent with findings of lower plasma tryptophan in women and higher values in men, potentially influenced by estrogen, progesterone, and testosterone (33). Nevertheless, the underlying cause of this sex-related difference in 5-HIAA values remains unclear, and additional studies are needed to assess its etiology.

The observation of a significant decrease in serum 5-HIAA after the initiation of SSA treatment adds to the usability of serum 5-HIAA in the follow-up of patients with CS. This is in line with Meyer and colleagues (20), who showed a reduction of plasma 5-HIAA after the initiation of SSA treatment in 46 patients with a NET. Our study does not provide evidence for the value of serum 5-HIAA measurements during PRRT. However, Zandee and colleagues showed a decrease in 24-hour urine 5-HIAA 6 months after PRRT (34), while we and others found a strong correlation between serum and 24-hour urine 5-HIAA (16, 17). This suggests the utility of serum 5-HIAA measurements in the follow-up of patients receiving PRRT.

Serum 5-HIAA measurements provide several benefits compared to 24-hour urine 5-HIAA, plasma 5-HIAA, and platelet serotonin measurements. When compared to a 24-hour urine collection, serum measurements provide a less troublesome and less sampling error-sensitive alternative. Additionally, serum provides a more stable alternative as serum 5-HIAA is stable for 7 days at room temperature (18), while 24-hour urine 5-HIAA tends to degrade after 24 hours (35). Also, Tohmola and colleagues (36) suggest that a 24-hour serotonin- and tryptophan-restricted diet might be sufficient for serum 5-HIAA measurements, which is shorter than the generally advised 48- to 72-hour diet for 24-hour urine 5-HIAA measurements (10). This contributes to the patient-friendliness of serum 5-HIAA measurements. Compared to serum 5-HIAA, platelet serotonin measurements offer the advantage of being unaffected by dietary serotonin intake (11). However, the limited saturation of serotonin in platelets, the limited stability of platelet serotonin, which requires these samples to be frozen or processed immediately after collection, and the small number of laboratories being able to perform this test limit their usability in the diagnosis and follow-up of patients with CS (9, 37). Compared to plasma 5-HIAA, serum 5-HIAA might give a more accurate representation of the total serotonin production as 5-HIAA stored in platelets is likely to be released during the clotting process (17).

To the best of our knowledge, this study includes the largest cohort of patients to date in describing the performance of 5-HIAA measurements for the diagnosis and follow-up of CS. Its main limitation is its retrospective nature, which could be accompanied by bias through incomplete reporting of CS symptoms in patient records. Also, we were unable to assess factors that influence serum and 24-hour urine 5-HIAA measurements, such as medication use and diet (10). Furthermore, 24-hour urine 5-HIAA measurements were analyzed using 2 different methods during the observed period: high-performance liquid chromatography and UPLC-MS/MS. However, given the strong correlation between the 2 methods (r = 0.99), the effects of using 2 different measuring methods may be considered negligible (38).

The use of the most recent definition of CS in this study resulted in a consistent and reproducible method for identifying patients with CS (4), in contrast to previous studies on plasma 5-HIAA for the diagnosis of CS, which did not use a clear definition (9, 17, 19). On the other hand, the use of this definition combined with the retrospective nature of this study raises uncertainty about the accurate identification of patients with CS, as CS-related symptoms may not have been correctly documented by the treating physician. This might have influenced both the diagnostic performance results and the found optimal cutoff values. Additionally, the use of this definition gives rise to a group of patients with NET and elevated 24-hour urine 5-HIAA values without CS-related symptoms, therefore not complying to the definition of CS. It is uncertain whether these patients have symptoms that are suppressed by SSA usage or whether these patients will develop CS-related symptoms in the future. Future prospective studies are needed to identify and characterize this group of patients.

In conclusion, serum 5-HIAA measurements are equivalent to 24-hour urine 5-HIAA measurements for the diagnosis of CS and may form a more reliable and convenient alternative. An optimal serum 5-HIAA cutoff value of 139.4 nmol/L was identified for the diagnosis of CS. The correlation of serum 5-HIAA with CS-related symptoms and tumor burden and its decrease after the initiation of SSA treatment indicate the usefulness of serum 5-HIAA in the follow-up of patients with CS.

## Data Availability

Some or all datasets generated during and/or analyzed during the current study are not publicly available but are available from the corresponding author on reasonable request.

## References

[1] Dasari A, Shen C, Halperin D, et al Trends in the incidence, prevalence, and survival outcomes in patients with neuroendocrine tumors in the United States. JAMA Oncol. 2017;3(10):1335‐1342. 28448665 10.1001/jamaoncol.2017.0589 PMC5824320

[2] White BE, Rous B, Chandrakumaran K, et al Incidence and survival of neuroendocrine neoplasia in England 1995-2018: a retrospective, population-based study. Lancet Reg Heal Eur. 2022;23:100510. 10.1016/j.lanepe.2022.100510 PMC9513765 36176500

[3] Halperin DM, Shen C, Dasari A, et al Frequency of carcinoid syndrome at neuroendocrine tumour diagnosis: a population-based study. Lancet Oncol. 2017;18(4):525‐534. 28238592 10.1016/S1470-2045(17)30110-9 PMC6066284

[4] Grozinsky-Glasberg S, Davar J, Hofland J, et al European neuroendocrine tumor society (ENETS) 2022 guidance paper for carcinoid syndrome and carcinoid heart disease. J Neuroendocrinol. 2022;34(7):e13146. 35613326 10.1111/jne.13146 PMC9539661

[5] Fijalkowski R, Reher D, Rinke A, et al Clinical features and prognosis of patients with carcinoid syndrome and carcinoid heart disease: a retrospective multicentric study of 276 patients. Neuroendocrinology. 2022;112(6):547‐554. 34348326 10.1159/000518651

[6] Ito T, Lee L, Jensen RT. Carcinoid-syndrome: recent advances, current status and controversies. Curr Opin Endocrinol Diabetes Obes. 2018;25(1):22‐35. 29120923 10.1097/MED.0000000000000376 PMC5747542

[7] Pearman TP, Beaumont JL, Cella D, Neary MP, Yao J. Health-related quality of life in patients with neuroendocrine tumors: an investigation of treatment type, disease status, and symptom burden. Support Care Cancer. 2016;24(9):3695‐3703. 27029477 10.1007/s00520-016-3189-z

[8] Beaumont JL, Cella D, Phan AT, Choi S, Liu Z, Yao JC. Comparison of health-related quality of life in patients with neuroendocrine tumors with quality of life in the general US population. Pancreas. 2012;41(3):461‐466. 22422138 10.1097/MPA.0b013e3182328045

[9] Carling RS, Degg TJ, Allen KR, Bax NDS, Barth JH. Evaluation of whole blood serotonin and plasma and urine 5-hydroxyindole acetic acid in diagnosis of carcinoid disease. Ann Clin Biochem. 2002;39(6):577‐582. 12564839 10.1177/000456320203900605

[10] Oberg K, Couvelard A, Delle Fave G, et al ENETS consensus guidelines for standard of care in neuroendocrine tumours: biochemical markers. Neuroendocrinology. 2017;105(3):201‐211. 28391265 10.1159/000472254

[11] Kema IP, Schellings AM, Meiborg G, Hoppenbrouwers CJ, Muskiet FA. Influence of a serotonin- and dopamine-rich diet on platelet serotonin content and urinary excretion of biogenic amines and their metabolites. Clin Chem. 1992;38(9):1730‐1736. 1382000

[12] Feldman JM, Lee EM. Serotonin content of foods: effect on urinary excretion of 5-hydroxyindoleacetic acid. Am J Clin Nutr. 1985;42(4):639‐643. 2413754 10.1093/ajcn/42.4.639

[13] Mashige F, Matsushima Y, Kanazawa H, et al Acidic catecholamine metabolites and 5-hydroxyindoleacetic acid in urine: the influence of diet. Ann Clin Biochem. 1996;33(1):43‐49. 8929065 10.1177/000456329603300106

[14] Corcuff J-B, Chardon L, El Hajji Ridah I, Brossaud J. Urinary sampling for 5HIAA and metanephrines determination: revisiting the recommendations. Endocr Connect. 2017;6(6):R87‐R98. 28566493 10.1530/EC-17-0071 PMC5527357

[15] Curtin F, Walker JP, Schulz P. Day-to-day intraindividual reliability and interindividual differences in monoamines excretion. J Affect Disord. 1996;38(2-3):173‐178. 8791186 10.1016/0165-0327(96)00011-0

[16] Wedin M, Mehta S, Angerås-Kraftling J, Wallin G, Daskalakis K. The role of Serum 5-HIAA as a predictor of progression and an alternative to 24-h urine 5-HIAA in well-differentiated neuroendocrine neoplasms. Biology (Basel). 2021;10(2):76. 33494283 10.3390/biology10020076 PMC7909826

[17] Adaway JE, Dobson R, Walsh J, et al Serum and plasma 5-hydroxyindoleacetic acid as an alternative to 24-h urine 5-hydroxyindoleacetic acid measurement. Ann Clin Biochem. 2016;53(5):554‐560. 26438520 10.1177/0004563215613109

[18] Tohmola N, Itkonen O, Sane T, et al Analytical and preanalytical validation of a new mass spectrometric serum 5-hydroxyindoleacetic acid assay as neuroendocrine tumor marker. Clin Chim Acta. 2014;428:38‐43. 24211728 10.1016/j.cca.2013.10.025

[19] de Mestier L, Savagner F, Brixi H, et al Plasmatic and urinary 5-hydroxyindolacetic acid measurements in patients with midgut neuroendocrine tumors: a GTE study. J Clin Endocrinol Metab. 2021;106(4):e1673‐e1682. 33382891 10.1210/clinem/dgaa924

[20] Meyer T, Caplin M, Khan MS, et al Circulating tumour cells and tumour biomarkers in functional midgut neuroendocrine tumours. J Neuroendocrinol. 2022;34(4):e13096. 35132704 10.1111/jne.13096 PMC9285714

[21] Tellez MR, Mamikunian G, O’Dorisio TM, Vinik AI, Woltering EA. A single fasting plasma 5-HIAA value correlates with 24-hour urinary 5-HIAA values and other biomarkers in midgut neuroendocrine tumors (NETs). Pancreas. 2013;42(3):405‐410. 23160483 10.1097/MPA.0b013e318271c0d5

[22] Ewang-Emukowhate M, Subramaniam K, Lam F, et al Plasma or serum 5-hydroxyindoleacetic acid can be used interchangeably in patients with neuroendocrine tumours. Scand J Clin Lab Invest. 2023;83(8):576‐581. 38112030 10.1080/00365513.2023.2286645

[23] van Haard PM . Chromatography of urinary indole derivatives. J Chromatogr. 1988;429:59‐94. 3062030 10.1016/s0378-4347(00)83867-0

[24] Agresti A, Coull BA. Approximate is better than “exact” for interval estimation of binomial proportions. Am Stat. 1998;52(2):119.

[25] Hanley JA, McNeil BJ. A method of comparing the areas under receiver operating characteristic curves derived from the same cases. Radiology. 1983;148(3):839‐843. 6878708 10.1148/radiology.148.3.6878708

[26] Youden WJ . Index for rating diagnostic tests. Cancer. 1950;3(1):32‐35. 15405679 10.1002/1097-0142(1950)3:1<32::aid-cncr2820030106>3.0.co;2-3

[27] Becker A, Schalin-Jäntti C, Itkonen O. Comparison of Serum and urinary 5-hydroxyindoleacetic acid as biomarker for neuroendocrine neoplasms. J Endocr Soc. 2021;5(8):bvab106. 34195530 10.1210/jendso/bvab106 PMC8237842

[28] Meijer WG, Kema IP, Volmer M, Willemse PH, de Vries EG. Discriminating capacity of indole markers in the diagnosis of carcinoid tumors. Clin Chem. 2000;46(10):1588‐1596. 11017936

[29] Lecoeur A, Sfeir RM, Gerard L, et al Serum and urinary 5-hydroxyindolacetic acid, serotonin, chromogranin A, and NT-proBNP for the detection of carcinoid heart disease. Eur J Endocrinol. 2024;191(6):570‐578. 39602485 10.1093/ejendo/lvae150

[30] Ortiz J, Artigas F, Gelpí E. Serotonergic status in human blood. Life Sci. 1988;43(12):983‐990. 2459577 10.1016/0024-3205(88)90543-7

[31] Houghton LA, Brown H, Atkinson W, et al 5-hydroxytryptamine signalling in irritable bowel syndrome with diarrhoea: effects of gender and menstrual status. Aliment Pharmacol Ther. 2009;30(9):919‐929. 19691669 10.1111/j.1365-2036.2009.04121.x

[32] Thijssen AY, Mujagic Z, Jonkers DMAE, et al Alterations in serotonin metabolism in the irritable bowel syndrome. Aliment Pharmacol Ther. 2016;43(2):272‐282. 26538292 10.1111/apt.13459

[33] Pais ML, Martins J, Castelo-Branco M, Gonçalves J. Sex differences in tryptophan metabolism: a systematic review focused on neuropsychiatric disorders. Int J Mol Sci. 2023;24(6):6010. 36983084 10.3390/ijms24066010 PMC10057939

[34] Zandee WT, Brabander T, Blažević A, et al Peptide receptor radionuclide therapy with 177Lu-DOTATATE for symptomatic control of refractory carcinoid syndrome. J Clin Endocrinol Metab. 2021;106(9):e3665‐e3672. 33942075 10.1210/clinem/dgab289 PMC8372632

[35] van Haard PM, Wielders JP, Wikkerink JB. Direct concurrent measurement of urinary vanillylmandelic acid, 5-hydroxyindoleacetic acid and homovanillic acid by HPLC. Three methodologies compared. Biomed Chromatogr. 1987;2(5):209‐215. 2466505 10.1002/bmc.1130020508

[36] Tohmola N, Johansson A, Sane T, Renkonen R, Hämäläinen E, Itkonen O. Transient elevation of serum 5-HIAA by dietary serotonin and distribution of 5-HIAA in serum protein fractions. Ann Clin Biochem. 2015;52(4):428‐433. 25249663 10.1177/0004563214554842

[37] Sanner JE, Frazier L, Udtha M. Effects of delayed laboratory processing on platelet serotonin levels. Biol Res Nurs. 2013;15(1):13‐16. 21859747 10.1177/1099800411416636 PMC3837525

[38] Clark ZD, Cutler JM, Frank EL. Practical LC-MS/MS method for 5-hydroxyindoleacetic acid in urine. J Appl Lab Med. 2017;1(4):387‐399. 33636811 10.1373/jalm.2016.021675

